# Implication of Vestibular Hair Cell Loss of Planar Polarity for the Canal and Otolith-Dependent Vestibulo-Ocular Reflexes in *Celsr1^–/–^* Mice

**DOI:** 10.3389/fnins.2021.750596

**Published:** 2021-11-01

**Authors:** François Simon, Fadel Tissir, Vincent Michel, Ghizlene Lahlou, Michael Deans, Mathieu Beraneck

**Affiliations:** ^1^Université de Paris, INCC UMR 8002, CNRS, Paris, France; ^2^Service d’ORL et de Chirurgie Cervico-Faciale Pédiatrique, AP-HP, Hôpital Necker-Enfants Malades, Paris, France; ^3^Institut de Neuroscience, Université Catholique de Louvain, Brussels, Belgium; ^4^College of Health and Life Sciences, Hamad Bin Khalifa University, Doha, Qatar; ^5^Institut de l’Audition, Institut Pasteur, INSERM, Paris, France; ^6^Institut de l’Audition/Institut Pasteur, Technologies et thérapie génique pour la surdité, Paris, France; ^7^Service d’ORL et de Chirurgie Cervico-Faciale Pédiatrique, APHP, Sorbonne Université, Hôpital Pitié-Salpétrière, Paris, France; ^8^Department of Neurobiology and Anatomy, University of Utah School of Medicine, Salt Lake City, UT, United States; ^9^Division of Otolaryngology, Department of Surgery, University of Utah School of Medicine, Salt Lake City, UT, United States

**Keywords:** vestibulo ocular reflex, planar cell polarity (PCP), vestibular system, CELSR1, mouse model, hair cell

## Abstract

**Introduction:** Vestibular sensory hair cells are precisely orientated according to planar cell polarity (PCP) and are key to enable mechanic-electrical transduction and normal vestibular function. PCP is found on different scales in the vestibular organs, ranging from correct hair bundle orientation, coordination of hair cell orientation with neighboring hair cells, and orientation around the striola in otolithic organs. Celsr1 is a PCP protein and a Celsr1 KO mouse model showed hair cell disorganization in all vestibular organs, especially in the canalar ampullae. The objective of this work was to assess to what extent the different vestibulo-ocular reflexes were impaired in Celsr1 KO mice.

**Methods:** Vestibular function was analyzed using non-invasive video-oculography. Semicircular canal function was assessed during sinusoidal rotation and during angular velocity steps. Otolithic function (mainly utricular) was assessed during off-vertical axis rotation (OVAR) and during static and dynamic head tilts.

**Results:** The vestibulo-ocular reflex of 10 Celsr1 KO and 10 control littermates was analyzed. All KO mice presented with spontaneous nystagmus or gaze instability in dark. Canalar function was reduced almost by half in KO mice. Compared to control mice, KO mice had reduced angular VOR gain in all tested frequencies (0.2–1.5 Hz), and abnormal phase at 0.2 and 0.5 Hz. Concerning horizontal steps, KO mice had reduced responses. Otolithic function was reduced by about a third in KO mice. Static ocular-counter roll gain and OVAR bias were both significantly reduced. These results demonstrate that canal- and otolith-dependent vestibulo-ocular reflexes are impaired in KO mice.

**Conclusion:** The major ampullar disorganization led to an important reduction but not to a complete loss of angular coding capacities. Mildly disorganized otolithic hair cells were associated with a significant loss of otolith-dependent function. These results suggest that the highly organized polarization of otolithic hair cells is a critical factor for the accurate encoding of the head movement and that the loss of a small fraction of the otolithic hair cells in pathological conditions is likely to have major functional consequences. Altogether, these results shed light on how partial loss of vestibular information encoding, as often encountered in pathological situations, translates into functional deficits.

## Introduction

Sensory hair cells play a key role in the vestibular system as they enable the transduction of mechanical head movements into the electrical signals that will inform the brain about the head movements and position in 3D space. This is made possible by the organization and polarization of the stereociliary bundle, a group of actin-made stereocilia on the apical hair cell surface that are arranged in rows of increasing height leading up to a microtubule-based kinocilium (Barr-Gillespie, 2015). Mechanical movement may deflect the bundle toward the kinocilium, placing tension on the tip-links, a think link connecting the tip of each stereocilium to the side of its taller neighbor and opening mechanoelectrical transducers (MET) channels, thus depolarizing the hair cell and sending an excitatory signal to the vestibular neurons (Nam et al., 2019). Movement away from the kinocilium conversely closes MET and results in an inhibitory stimulus. Each hair cell therefore has a specific directional sensitivity that corresponds to its polarity axis (Shotwell et al., 1981). In both the semicircular canals (SCC) and otolithic organs, hair cells are arranged and coordinated according to their neighboring cells during vestibular morphogenesis (Yang et al., 2017). In the SCC, the stereociliary bundles are all orientated in the same direction parallel to the SCC axis and are thus all stimulated at the same time. In the utricular and saccular maculae, orientation of the hair cells cover 360° and are all organized in a mirror-like fashion around a cell boundary called Line of Polarity Reversal (LPR), which runs along the center of the macula in close proximity to the striolar region (Deans, 2013; Yang et al., 2017).

This complex organization, termed planar cell polarity (PCP), may be found on different scales: intra-cellular scale within the stereociliary bundle, inter-cellular scale where hair cell orientation depends on the orientation of neighboring hair cells, and in the otolithic organs on a macular scale, as each head movement combines an excitatory and inhibitory stimulus on either side of the LPR (Deans, 2013). PCP proteins are key in all vertebrate systems to enable cell communication and coordination between hair cells and supporting cells, as well as between supporting cells (Deans, 2013; Tissir and Goffinet, 2013; Hakanen et al., 2019), notably in the inner ear (Tarchini and Lu, 2019). In humans, the CELSR1 molecule (Cadherin EGF LAG Seven-pass G-type Receptor 1), a PCP protein, has been linked to neural tube defects and caudal agenesis (Allache et al., 2012; Robinson et al., 2012), although no specific vestibular function anomaly has been reported.

To better study the effect of the Ceslr1 molecule, *Celsr1^–/–^* mice have been developed introducing a frameshift that leads to a premature stop preventing translation of the cytoplasmic domain. Most *Celsr1* knocked-out (KO) mice are not viable due to neural tube defects: 20% die *in utero* and more than half of the remaining die before weaning (Ravni et al., 2009). Indeed, Celsr1 is a protein involved in PCP formation but is also linked to several severe defects in neurological development. Various *Celsr1* mouse mutants also present with severe neural tube defects ranging from craniorachischisis to loop-tails (Greene et al., 2009), behavioral impairment (Boucherie et al., 2018), alterations of skin hair pattern (Ravni et al., 2009), endothelial valve formation (Tatin et al., 2013), and oviduct development (Shi et al., 2014).

**FIGURE 1:**
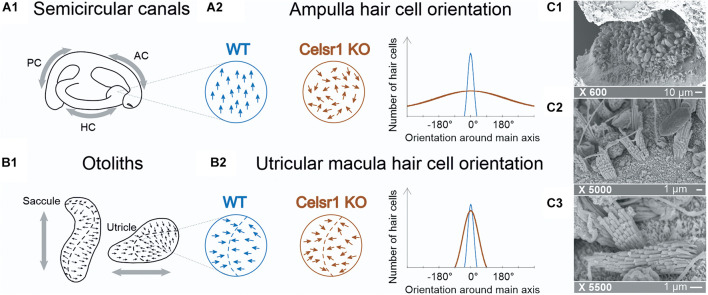
Hair cell orientation of semicircular canal ampullae and otolithic maculas in wild-type and *celsr1^− /−^* mice. In semicircular canals, the ampullae **(A1)** are stimulated following a rotation, whereas **(B1)** the utricule and saccule are stimulated following translational movements. The organization of hair cells in the ampulla **(A2)** are normally all orientated in the same direction, whereas in *celsr1^− /−^* mice, 80% of hair cells are misorientated. The organization in the macula **(B2)** is more complex with hair cells orientated toward the striola (in the utricule shown here), and moderate misorientation in *celsr1^− /−^* mice. Hair cell orientation of wild type (WT) and *celsr1^− /−^* are schematically represented based on results from Duncan et al. (2017). Transmission electronic microscope of the saccular macula of a KO mouse. The × 600 **(C1)** image shows an overall maintained saccular structure with otoconia and sensory hair cells beneath; **(C2)** × 5,000 shows mostly stereociliary bundles and **(C3)** × 5,500 shows a clear misalignment of two neighboring hair cells, confirming the previous study (Duncan et al., 2017).

Concerning the labyrinth, auditory hair cell misorientation has been reported (Curtin et al., 2003). Those *Celsr1* KO mice that survive were not found to have any auditory impairment, which may be due to compensation by other *Celsr* genes in the KO that does not occur in other *Celsr1* mutant lines (Tissir and Goffinet, 2013; Duncan et al., 2017). The mice, however, presented with typical vestibular postural and locomotor disorders such as head bobbing, circling, and spinning when suspended by the tail (Curtin et al., 2003; Duncan et al., 2017). Although associated with vestibular malfunction, these postural impairments are largely non-specific and do not allow us to distinguish SCC- from otolith-based deficits (Beraneck et al., 2014). Hence, immunofluorescent imaging of the vestibular organs showed that in the absence of Celsr1, stereociliary bundles were misoriented relative to their neighbors, especially in the SCC (Figure 1A). On the other hand, the orientation at the level of the maculae was found to be only mildly affected (Duncan et al., 2017; Figure 1B). To which extent the functionality of vestibular-dependent reflexes relies on the precise orientation of the population of hair cells in the ampullae and maculae remains to our knowledge completely unexplored.

The objective of this study was therefore to precisely quantify in the *Celsr1* KO adult mice how the differential disorganization of the vestibular hair cells polarity affects the canalar- and otolithic-dependent vestibulo-ocular reflexes.

## Materials and Methods

Animals were used in accordance with the European Communities Council Directive 2010/63/EU. All efforts were made to minimize suffering and reduce the number of animals included in the study. All procedures were approved by the ethical committee for animal research of the University of Paris. Animals from the *Celsr1* KO line (Ravni et al., 2009) were provided by the Université Catholique de Louvain. 10 *Celsr1^–/–^* and 10 littermate control mice were analyzed.

To perform pupil position recording with a fixed head, a head post was implanted at least 48 h before vestibular exploration to the skull (Beraneck and Cullen, 2007; Beraneck et al., 2012; França de Barros et al., 2019). All eye movement recordings were made in the dark using an infrared video system (ETL-200, ISCAN, Burlington MA), recording pupil, and corneal reflection (CR) position (Beraneck and Cullen, 2007; Beraneck and Lambert, 2009; Beraneck et al., 2012; Beraneck and Idoux, 2012). Eye movements were recorded using non-invasive video-oculography (Stahl et al., 2000). The experimental setup, apparatus, and methods of data acquisition were similar to those described previously (Beraneck et al., 2012; Carcaud et al., 2017; Idoux et al., 2018). Briefly, mice were head-fixed at a ∼30° nose-down position to align the horizontal canals with the yaw plane (Calabrese and Hullar, 2006). Myosis was induced with topical 2% pilocarpine applied 10 min before experimentation. Recorded eye and head position signals were sampled at 1 kHz, digitally recorded (CED power1401 MkII) using Spike 2 software and later exported into the Matlab programming environment for off-line analysis (Matlab, The MathWorks). Videonystagmography recorded spontaneous eye movement without vestibular stimulation and eye movement with the three sequential following stimulations: sinusoidal rotation for horizontal angular vestibulo-ocular reflex (aVOR), off-vertical axis rotation (OVAR) for maculo-ocular reflex (MOR), bias and modulation and static and dynamic head tilt roll for the ocular counterroll (OCR) (Beraneck et al., 2012; Romand et al., 2013; Simon et al., 2020).

First, aVOR was tested during horizontal sinusoidal rotation of the turntable (at 0.2, 0.5, 0.8, 1, and 1.5 Hz; peak velocity 30°/s), analyzing gain and phase. The gain was the ratio between the velocity of the eye (response) and head (stimulus) rotations. Since the animal was head-fixed to the rotating table, head movements and table movements were identical. The phase was the temporal shift between the eye and table rotations, expressed in degrees as ratio of the sinusoidal cycle (2 pi). Details for gain and phase calculation were reported in Carcaud et al. (2017). Values with VAF (Variance-accounted-for) under 0.5 were discarded (Beraneck and Cullen, 2007). During OVAR test (Hess and Dieringer, 1990), axis of rotation was tilted from the vertical by 17°. Rotations were performed at constant speed (50°/s) for at least 10 rotations both in clockwise (CW) and counterclockwise (CCW) directions. During rotations, the velocity of horizontal slow phases is modulated around a constant bias. All methods and analysis during OVAR are similar to those in Beraneck et al., 2012. The MOR corresponds to an otolithic response, but also critically depends on an efficient central vestibular system (Hess and Dieringer, 1990; Beraneck et al., 2012). The static OCR tests more specifically the static utricular function. Vertical pupil position according to the head tilt angle was measured first with the mouse maintained at a 0° horizontal position. The platform was then tilted into different roll positions, at 10°, 20°, 30°, and 40° alternatively to the right and to the left. Measurements were made in a static position during at least 15 s to identify the stable pupil position. The vertical eye angle was then calculated from the raw vertical CR and pupil position (Oommen and Stahl, 2008). The OCR gain was calculated corresponding to the slope of a linear regression of both variables (vertical eye angle and head tilt degree). Dynamic roll head tilt was also tested during sinusoidal roll motion at 0.5 Hz from left to right at three different roll angles: −10° to 10°, −20° to 20°, and −30° to 30° corresponding to three different roll amplitudes: 20°, 40°, and 60°. Maximal amplitude of the sinusoidal vertical pupil position was calculated for each condition and dynamic _tilt_VOR gain was calculated corresponding to the amplitude of the vertical eye position on amplitude of the roll rotation. Finally, angular velocity steps in the horizontal plane (hsteps) were performed at a speed of 50°/s. The horizontal slow phase velocity decay was fitted to an exponential curve [*f*(*x*) = *a*^∗^exp(*b*^∗^*x*)] and the time constant *τ* was then calculated as *τ* = −1/b. Gain was calculated from the peak slow phase velocity on table velocity. The time constant of the slow phase exponential velocity decay and gain was calculated for per-rotatory and post-rotatory nystagmus for CW and CCW rotations. CCW per-rotatory and CW post-rotatory values, and CCW post-rotatory and CW per-rotatory values, were combined to assess left and right vestibular functions, respectively. Directional preponderance was calculated using the Jongkees formula and gain value:*DP* = *Leftgain*−*RightgainLeftgain*+*Rightgain* The “saccade main sequence” (Bahill et al., 1975) was analyzed by comparing the relationship between fast phases’ peak velocity, duration, and amplitude (Stahl, 2008) to assess the ability of the ocular motor system to generate force (Leigh and Zee, 2015) and integrity of premotor oculomotor pathway (Gibaldi and Sabatini, 2021). For each individual, at least 15 fast phases with peak velocity above 80°/s produced during the OVAR test were analyzed. Onsets and offsets were defined using a ± 20°/s gaze velocity criterion (Beraneck and Cullen, 2007).

Once vestibular exploration was complete, mice were euthanized. In three mice (two Celsr1 KO and one WT), temporal bones were dissected and an opening was made in the apex of the cochlea before fixation in 2.5% glutaraldehyde in cacodylate buffer pH 7.4 at 4°C for 2 h. Vestibular organs were later microdissected and processed for scanning electronic microscopy by alternating incubations in 1% osmium tetroxide and 0.1 M thiocarbohydrazide (OTOTO), as previously described (Furness et al., 2008), to check the disorganization of the morphology of hair bundles in the sensory cells (Figure 1C).

Statistical analysis was made using XLstats (Addinsoft, New York, NY). All data are reported as mean and standard deviation. Normal distribution of values was verified using the Kolmogorov–Smirnov test. Two-way ANOVA was used to compare aVOR gain and phase (mouse type and frequency) and the parameters of the Saccade Main Sequence (mouse type and parameters). One-way ANOVA was used to compare static and dynamic roll head tilt amplitudes. *Post-hoc* comparisons were performed where appropriate using the Tukey HSD test. Student’s *t*-test (or Wilcoxon if appropriate) was used for MOR bias and modulation static OCR and dynamic _tilt_VOR. For fast phase analysis, a detection of outliers was performed using Routs method and all statistics on regression lines were performed using GraphPad Prism software. Values of *p* < 0.05 were considered significant.

## Results

### Behavioral and Spontaneous Observations

A total of 10 *Celsr1^–/–^* (KO mice) and 10 control littermates/wild type (WT) were tested at adult age, and the characteristics of both groups are reported in Table 1. All KO mice had abnormal swimming behavior, circling, and head tilt, but none of them drowned.

**TABLE 1:** Characteristics of mice tested.

**Type of mice**	**Number of mice**	**Number of males**	**Age (weeks) when tested**	**Number of loop-tails**
*Celsr1^–/–^*	10	2	13 ± 9	6
WT	10	6	15 ± 9	0

*Age reported as mean ± standard deviation.*

Videonystagmography recording eye stability in the dark without any vestibular stimulation showed abnormal eye movements in the KO group only. Five mice had spontaneous horizontal nystagmus with the rapid eye movement always beating in the same direction. The five other mice had spontaneous horizontal nystagmus, which could beat in either direction. The KO mice had 10.8 ± 7 spontaneous nystagmus beating per minute (regardless of direction). The presence of spontaneous horizontal nystagmus in all KO mice shows that the disorganization of the vestibular hair cells planar polarity probably affects the balance between the mass discharge within bilateral vestibular complex, which is a major determinant of the stability of gaze in the horizontal plane.

### Canal-Dependent Vestibulo-Ocular Reflex Assessment

**FIGURE 2:**
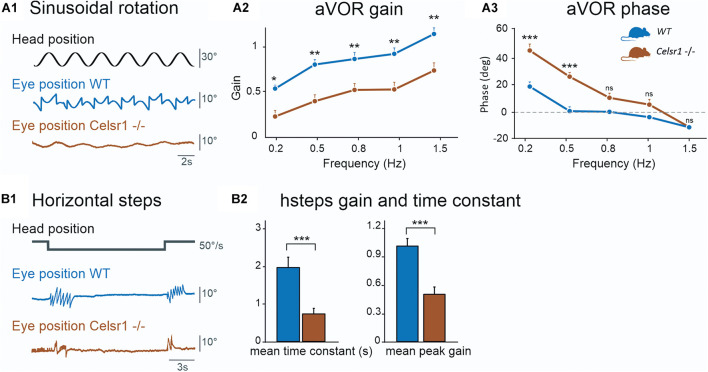
Semicircular canal function. The horizontal semicircular canal (SCC) was tested. Horizontal sinusoidal rotation **(A)** tested for horizontal aVOR. Traces in **(A1)** show head position (corresponding to table position) and horizontal eye movement. KO mice show clear reduction in the amplitude of the eye movement. aVOR gain **(A2)** for maximum 30°/s velocity showed reduced gain for all frequencies of stimulation. aVOR phase **(A3)** for maximum 30°/s velocity showed increased phase for all frequencies of stimulation except 1.5 Hz. Horizontal steps **(B1)** at 50°/s were performed. Traces show head position and horizontal eye position, during per-rotatory nystagmus at the start of a CCW stimulation. In this example trace, only two fast phases can be identified at velocity changes for the KO mouse compared to seven for the WT. Time constant (**B2**, left) and peak gain (**B2**, right) are both decreased for Celsr1 KO mice, for hsteps at 50°/s, for per- and post-rotatory nystagmus during CW and CCW steps. *aVOR* angular vestibulo-ocular reflex, *CW* Clockwise, *CCW* Counterclockwise, *hsteps* horizontal steps, *WT* wild type. *p < 0.05; **p < 0.01; ***p < 0.001.

The amplitude of the eye movements evoked by sinusoidal horizontal rotations was reduced in KO compared to controls (Figure 2A1). The gain and phase of the angular horizontal vestibulo-ocular reflex are illustrated in Figures 2A2,A3 and values are reported in Table 2. KO mice had significantly lower gain over all frequencies [two-way ANOVA model, *F*(9, 100) = 17.1, *p* < 0.001], with a reduction of about 50% of the amplitude of eye movements in all tested conditions. This deficit was accompanied with a significant phase lead observed in the lower frequencies only [two-way ANOVA model, *F*(9, 100) = 37.9, *p* < 0.001].

**TABLE 2:** Horizontal angular vestibulo-ocular reflex.

**Frequency**	**0.2 Hz**	**0.5 Hz**	**0.8 Hz**	**1 Hz**	**1.5 Hz**
**aVOR gain**					
*Celsr1^–/–^*	0.24 ± 0.22	0.42 ± 0.24	0.55 ± 0.21	0.55 ± 0.27	0.77 ± 0.27
WT	0.56 ± 0.13	0.84 ± 0.19	0.91 ± 0.24	0.97 ± 0.22	1.20 ± 0.22
*p*	***0.044***	***0.002***	***0.015***	***0.002***	***0.002***
**aVOR phase**					
*Celsr1^–/–^*	48.7 ± 16.2	28.0 ± 10.4	11.4 ± 10.4	6.1 ± 11.7	−11.6 ± 3.8
WT	20.3 ± 11.5	1.0 ± 10.7	0.4 ± 5.7	−3.8 ± 4.7	−11.8 ± 6.6
*p*	***<0.001***	***<0.001***	*0.266*	*0.423*	*1*

*All values are represented as mean ± SD (standard deviation). Statistical significance of the difference between wild type (WT) and Celsr1 KO mice is shown, significant values are in bold (normal distribution). aVOR angular Vestibular-ocular reflex; *p* values shown in italic.*

Function of the horizontal aVOR was further tested during velocity steps at 50°/s. At the onset of the movement, horizontal eye movements were typically observed as a succession of compensatory slow phases interrupted by fast phases that recentered the eye. In WT mice, the responses lasted several seconds while it only lasted 1–2 s in KO (Figure 2B1). Hsteps results are reported in Table 3. Overall, peak-velocity gain and time constant were both significantly reduced in KO mice (Figure 2B2). This confirms the general hypofunction of the canal-dependent aVOR. To further quantify asymmetry of the responses, results are reported according to side of stimulation (Table 3). Gains and time constants were reduced in *Celsr1* KO mice in both ears. Directional preponderance (Table 3, see section “Materials and Methods”) was significantly increased in KO mice compared to WT, regardless of the direction. Thus, although no side stood out, there was an overall increased instability and variability in the preponderance of the deficit in the KO mice, compatible with the gaze instability observed at rest, which was not always in the same direction. No statistical correlation was found between the directional preponderance and nystagmus direction. During both sinusoidal angular rotation or horizontal steps, compensatory eye movements were restricted to the horizontal plane, with minimal vertical component, as observed in WT mice, suggesting that the spatial tuning of the canal-dependent VOR was unaffected by the disorganization of hair cell polarity.

**TABLE 3:** Horizontal steps.

	**Left gain**	**Right gain**	**Left τ**	**Right τ**	**Overall gain**	**Overall τ**	**Directional preponderance**
*Celsr1^–/–^*	0.48 ± 0.22	0.53 ± 0.31	0.70 ± 0.50	0.81 ± 0.50	0.51 ± 0.25	0.76 ± 0.48	0.18 ± 0.11
WT	0.97 ± 0.22	1.08 ± 0.31	2.18 ± 0.82	1.88 ± 1.05	1.03 ± 0.26	2.03 ± 0.90	0.06 ± 0.06
*p*	*−*	*−*	*−*	*−*	***<0.001***	***0.001***	***0.009***

*All values are represented as mean ± SD (standard deviation). Overall gain and τ values correspond to the mean value of CCW and CW per- and post-rotatory values. τ is in seconds. Absolute values were used to calculate directional preponderance mean. Statistical comparison was made using Student’s t-test (normal distribution). τ time constant. Significant values are shown in bold.*

### Otolith-Dependent Vestibulo-Ocular Reflex Assessment

**FIGURE 3:**
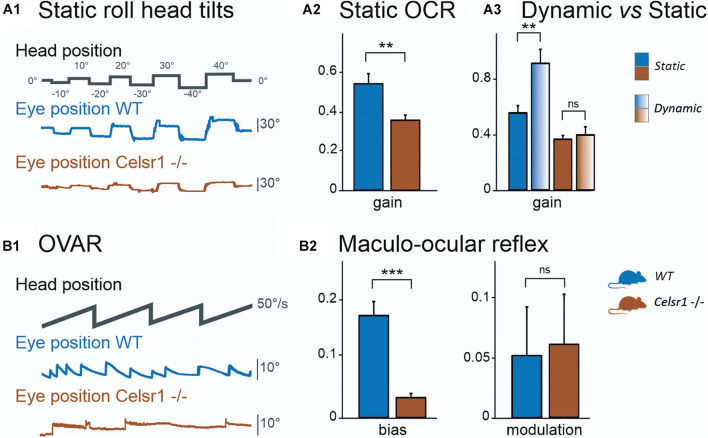
Otolithic system function. Static roll head tilts **(A1)** were performed to determine OCR testing utricular function. Traces show head position (corresponding to table position) and vertical eye movement. KO mouse had preserved function with about 1/3 poorer gain. Vertical eye position in degrees was calculated for OCR according to roll head tilt degree. The static left eye pupil position was measured; tilts with positive degrees were toward the left side and those with negative degrees were toward the right side. OCR gain **(A2)** was significantly reduced in KO mice. The dynamic (gradient fill) head tilt gain was compared to the static (solid fill) OCR gain **(A3)**. In KO mice, the amplitude was not significantly changed between static and dynamic stimulations, whereas it was in WT mice. The Maculo-ocular reflex was studied with the off-vertical axis rotations **(B1)** at 50°/s. Traces show head position and horizontal eye position, during CW stimulation. KO mouse in this example has poor response, seen as the absence of the MOR nystagmus. We report MOR bias (**B2**, left), which is significantly reduced in KO mice, and modulation (**B2**, right), which is similar in both groups. *CW* Clockwise, *CCW* Counterclockwise, *MOR* Maculo-ocular reflex, *OCR* Ocular counterroll, *OVAR* Off-vertical axis rotation, *WT* wild type. *p < 0.05; **p < 0.01; ***p < 0.001.

To specifically test the otolith-dependent vestibulo-ocular reflexes, WT and KO mice were tested using roll head tilt (Simon et al., 2020), which activates the ocular counter rotation reflex (OCR), and during OVAR, which activates the MOR (Beraneck et al., 2012). Results of both tests are illustrated in Figures 3A,B, respectively. Static OCR gain and MOR bias and modulation values are reported in Table 4.

**TABLE 4:** Static OCR and OVAR–maculo-ocular vestibular reflexes.

**MOR**	**Static ocular counterroll**			**Off-vertical axis rotation**			
	**40° left**	**40° right**	**Gain**	**CCW bias**	**CW bias**	**Overall bias**	**Overall gain mod.**
*Celsr1^–/–^*	14.7 ± 8.2	*−*16.9 ± 8.1	0.37 ± 0.09	*−*0.02 ± 0.03	0.04 ± 0.04	0.03 ± 0.03	0.06 ± 0.04
WT	17.7 ± 7.4	*−*29.0 ± 13.0	0.56 ± 0.17	*−*0.17 ± 0.10	0.17 ± 0.11	0.17 ± 0.10	0.05 ± 0.04
*p*	*−*	*−*	***0.008***	*−*	*−*	*<* ***0.001***	*0.38*

*All values are represented as mean ± SD (standard deviation). OCR is reported as the left eye vertical angle at 40° static head tilt to the left and to the right and gain (slope value of vertical eye angle and head tilt degree). Maculo-ocular reflex during OVAR is reported with the bias value, during CCW and CW stimulations, and overall bias mean. MOR modulation is also reported (overall CW and CCW values). Statistical comparison was made using Student’s t-test (normal distribution).*

*CCW counterclockwise, CW clockwise, MOR maculo-ocular reflex.*

*Significant values are shown in bold.*

Figure 3A1 illustrates the vertical eye movements observed during the static roll head tilt. The amplitude of the OCR responses was clearly reduced in KO mice compared to controls. The static OCR gain in KO mice was reduced by about 1/3 compared to WT mice, with mean amplitude gain (vertical eye amplitude/tilt amplitude) of 0.34 ± 0.07 vs. 0.57 ± 0.20, *p* = 0.062, for KO and WT mice, respectively (Figure 3A2). The responses of dynamic OCR were then tested in response to dynamic head roll tilts performed as 0.5-Hz sinusoidal rotations in the roll axis. The gain of dynamic OCR was of 0.40 ± 0.19 vs. 0.93 ± 0.31, *p* < 0.001, for KO and WT mice, respectively [ANOVA model *F*(3, 40) = 17.5, *p* < 0.001]. No significant difference was found in the responses between static and dynamic OCR in the KO mice, whereas the responses were significantly better during dynamic OCR than static OCR in the WT mice (Figure 3A3).

The responses of WT and KO mice to OVAR stimulation are presented in Figure 3B1 and Table 4. Again, a clear hypofunction was found in KO mice, as an absence of the OVAR-evoked nystagmus (Figure 3B2). Quantification revealed an absence of MOR in KO mice during both clockwise and counterclockwise rotations. Overall, the results of the head roll and OVAR tests reveal major deficits in the otolith-dependent reflexes. Both OCR and MOR appeared significantly impaired in KO compared to WT. Altogether, these results show that a mild impairment in the orientation of the hair cells on the maculae leads to severe dysfunction affecting the otolith-dependent vestibulo-ocular reflexes.

### Assessment of the Ocular Motor Pathway

**FIGURE 4:**
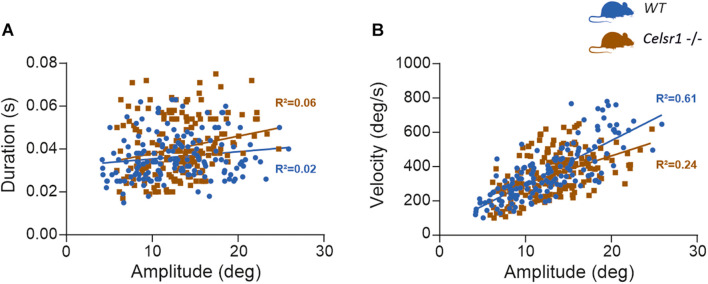
Saccade main sequence. At least 15 fast phases generated by WT (*n* = 10) and KO mouse (*n* = 10) during OVAR stimulation were analyzed, for a total 159 and 186 fast phases, respectively. The amplitude, duration, and peak velocity were quantified using a 20°/s threshold for saccade onset and offset (Beraneck and Cullen, 2007). **(A)** Shows the relation between amplitude and duration. For WT, linear regression parameters were: *Y* = 0.0003*x* + 0.032; *R*^2^ = 0.024; *F*(1, 157) = 3.93, *p* = 0.049. For KO, linear regression parameters were: *Y* = 0.0008*x* + 0.030; *R*^2^ = 0.055; *F*(1, 184) = 10.64, *p* = 0.001. Regression were not significantly different [*F*(1, 341) = 2.53, *p* = 0.113]. **(B)** Shows the relation between amplitude and velocity. For WT, linear regression parameters were: *Y* = 25.38*x* + 44.02; *R*^2^ = 0.607; *F*(1, 155) = 239.3, *p* < 0.0001. For KO, linear regression parameters were: *Y* = 16.08*x* + 140.7; *R*^2^ = 0.242; *F*(1, 184) = 58.59, *p* < 0.0001. Regression were significantly different [*F*(1, 339) = 12.24, *p* < 0.001].

The Celsr1 KO mouse is a model of planar cell polarization loss in the vestibular organs. However, several other abnormalities have been reported in this strain (Curtin et al., 2003; Tatin et al., 2013; Shi et al., 2014; Boucherie et al., 2018), which clearly demonstrates deficits in the central nervous system. To determine whether the reduced responses recorded during VOR tests could relate to a deficit in the ability of the ocular motor system to generate force (Leigh and Zee, 2015), we quantified the so-called “saccade main sequence” (Bahill et al., 1975; Gibaldi and Sabatini, 2021). The quantification of the peak velocity, amplitude, and duration of fast phases produced during vestibular stimulation (OVAR test) demonstrated no statistically significant difference between WT and KO (Table 5). There was no significant difference in the relationship between amplitude and duration either (*p* = 0.11, Figure 4A). As previously reported in several species (Stahl, 2008), horizontal fast phases exhibited a rather linear relationship between velocity and amplitude for WT mice (*R*^2^ = 0.61, *p* < 0.001, Figure 4B); KO mice demonstrated a less robust relationship (*R*^2^ = 0.24, *p* < 0.001), with a reduced slope compared to WT (*p* < 0.001). While informative, this analysis is based on oculomotor responses generated by vestibular stimulation. Specific oculomotor tests are needed to confirm whether KO mouse show normal or altered ocular motor function for eye movements evoked via other means (optokinetic nystagmus for example).

**TABLE 5:** Saccade main sequence.

	**Peak velocity (°/s)**	**Amplitude (°)**	**Duration (s)**
*Celsr1^–/–^*	353.3 ± 124.4	13.2 ± 3.8	0.041 ± 0.013
WT	370.0 ± 166.6	12.7 ± 4.8	0.036 ± 0.010
*p*	*0.44*	*1*	*1*

*All values are represented as mean ± SD (standard deviation). For each group, a minimum of 15 fast phases were analyzed per mice; n = 186 and n = 159 for Celsr1^–/–^ and WT, respectively. Statistical comparison was made using a two-way ANOVA model [two-way ANOVA model, F(5, 1035) = 821.3, p < 0.001] with Tukey post-hoc tests (normal distribution).*

## Discussion

It has previously been shown that in *Celsr1* KO mice, SCC ampullar hair cells were particularly disorganized, with approximately less than 20% of hair cells correctly oriented, whereas the loss of polarity was milder in the otolithic organs with approximately more than 80% of hair bundles in the appropriate direction (Figure 1; Duncan et al., 2017). This disorganization was confirmed in our KO mouse population. Although morphological, locomotor, and postural behavioral studies had already been undertaken, the effect of stereociliary bundle polarity loss on the capacity of the vestibular system to encode precise directional signals during head movements had not yet been studied.

It must be noted that the mutated *celsr1* not only affects the peripheral vestibular system but also has multiple other consequences including severe neurological defects such as neural tube defects and caudal agenesis (Curtin et al., 2003; Boucherie et al., 2018). Thus, it seems likely that *Celsr1* KO mice have altered central processing due to major developmental perturbation in addition to altered PCP formation in the vestibular periphery. This should be kept in mind when analyzing the results, as all differences between KO and WT mice may not simply be due to peripheral organ disorganization but may be partly linked to central pathway and/or premotor ocular anomalies.

In our study, we found that the canalar function was severely altered, with aVOR gain in KO approximately half of that of WT mice on all frequencies. A statistically significant phase lead was, however, only found in the lower frequencies at 0.2 and 0.5 Hz, and became progressively identical to WT phases at high frequencies (>1.5 Hz). This suggests that loss of cell polarity impairs the amplitude of compensatory eye movement at all frequencies, and the timing of the aVOR more specifically at lower frequencies. A hypothesis to explain this result would be that, angular accelerations being lower at those frequencies, the misoriented hair cells are likely not stimulated or weakly stimulated, leading to an abnormally decreased population encoding of the movement. This probably impairs the capacity of central vestibular neurons to appropriately encode the amplitude and timing of the head movement. In higher frequencies, a larger proportion of hair cells would be stimulated at the onset of movement, facilitating the event detection of movement and enabling a VOR response with a normal phase. Whichever the frequency, the reduced number of correctly aligned stereocilia bundles depolarized during canalar stimulation could explain the reduced gain over the entire frequency range tested. This was confirmed by analyzing the mean peak gain during the hsteps stimulation, in response to a transient angular stimulation. Indeed, a 0.51 vs. 1.03 gain was measured, showing the loss of approximately half of the canalar gain, yet unexpectedly high considering that more than 80% of ampulla hair cells were disorganized. Overall, it should be noted that despite an extensive disorganization of the hair cell orientation in the ampullae, the signals originating from one SCC could be centrally interpreted as a directional activation in the plane of the canal, thus preserving the directionality of the generated eye movement. Overall, these results suggest that a major loss of information encoding at the level of the semicircular canal, as probably occurs in many inner ear pathologies such labyrinthitis or ototoxicity (Cassel et al., 2019), can partly be compensated by the central vestibular complex. It further suggests that the decoding of directionality of the movement is mostly accounted for by the orientation of the semicircular canals and not by hair cell orientation *per se*.

Otolithic-dependent VOR were significantly decreased in the *Celsr1* KO mice; however, the residual function was sufficient to maintain a static OCR gain at approximately two-thirds of WT mice gain, which could explain why none of the *Celsr1* KO mice drowned in our study. Considering the minor loss of hair cell polarization in the utricle previously reported morphologically (Duncan et al., 2017), functional loss may be considered excessive compared to the ampulla. This may be due to the fact that the otolithic system contains less redundancy in hair cell direction (as hair cells tend to follow the axis of the striola perpendicularly), compared to the ampulla where all hair cells are normally orientated in the same direction (Deans, 2013). This result demonstrates that the spatial organization of the hair cells in the maculae is a critical factor allowing the precise decoding of the spatial directionality of the head movements by central vestibular structures. Thus, in the otolithic system, a small proportion of hair cells misoriented may incur major functional impairment.

A number of other tests assessed more integrated and complex vestibular functions, such as the velocity storage (which requires SCC function and the integrity of a central vestibular and cerebellar neural circuit), MOR (the bias corresponding to a complex otolithic response, but which also requires the integrity of canalar and velocity storage system), and the dynamic roll head tilt (which recruits both utricular and vertical canal function, with an increase in gain compared to the static tilt OCR (Maruta et al., 2001)). No test was able to specifically assess vertical SCC function, although we may expect from this last test that the reduction in vertical SCC function might be comparable to that of horizontal SCC function, as a comparable hair cell disruption has been found in both (Duncan et al., 2017). Differences between KO and WT mice were strongly significant concerning hsteps time constant and peak gain, as well as MOR bias and dynamic roll head tilt. This suggests that the central vestibular complex is not able to compensate for the accumulated peripheral deficits by integrating information originating from different vestibular organs.

## Conclusion

Previous studies demonstrated that during development, the acquisition of optimal canal and otolith-based responses are mutually dependent, that is, a deficit in one set of organs might affect the maturation of the other. In otolith-deficient mice that lack otolith-based reflexes, the spatial tuning of the aVOR was thus found to be impaired (Beraneck et al., 2012). Similarly, early alteration of semicircular canals was demonstrated to affect the translational, otolithic-dependent, VOR in *Xenopus laevis* (Branoner and Straka, 2015, 2018). One objective of our study was thus to confirm which characteristics (amplitude of response, timing of the response, and directional tuning) of the canal and otolith-dependent VOR were affected by the peripheral vestibular hair cell loss of planar polarity.

This study first confirms the previous morphological results (Duncan et al., 2017), showing that in *Celsr1* KO mice, vestibulo-ocular functional reflexes depending on canal or otolith organs are both impaired. Overall, the major ampullar disorganization led to a reduction, but not to a complete loss of angular coding capacities, and no abnormal spatial tuning. On the other hand, mildly disorganized otolithic hair cells were associated with a marked hypofunction. These results therefore suggest that the highly organized polarization of otolithic hair cells is a critical factor for the accurate encoding of the head movement and that the loss of a small fraction of the otolithic hair cells is likely to have major functional consequences. These results shed light on how partial loss of vestibular information encoding, as often encountered in pathological situations, translates into functional deficits.

Although *CELSR1* missense or dinucleotide repeat mutations have been associated with neural tube defect studies (Allache et al., 2012; Robinson et al., 2012; Lei et al., 2014; Zhan et al., 2016), vestibular function in these patients has never been reported. Considering their neurological background, exploration of vestibular function may not be sufficiently considered in patients with neural tube defects who present with posture, reaching, or orientation issues. Bearing in mind our results, we believe that clinical research studies should assess vestibular function in patients with neural tube defects and especially with *CELSR1* mutations, as vestibular disorders may be diagnosed and appropriate rehabilitation may be prescribed.

## Data Availability Statement

The raw data supporting the conclusions of this article will be made available by the authors, without undue reservation.

## Ethics Statement

The animal study was reviewed and approved by Ethical Committee for Animal Research of the University of Paris.

## Author Contributions

FS and MB carried out the experiment. FS wrote the manuscript with support from FT, VM, GL, MD, and MB. FT produced the *Celsr1* knockout mice. MD and FT helped supervise the project. MB and MD conceived the original idea. MB supervised the project. All authors edited the manuscript and approved its content.

## Conflict of Interest

The authors declare that the research was conducted in the absence of any commercial or financial relationships that could be construed as a potential conflict of interest.

## Publisher’s Note

All claims expressed in this article are solely those of the authors and do not necessarily represent those of their affiliated organizations, or those of the publisher, the editors and the reviewers. Any product that may be evaluated in this article, or claim that may be made by its manufacturer, is not guaranteed or endorsed by the publisher.
